# Trauma-informed care (TIC) best practices for improving patient care in the emergency department

**DOI:** 10.1186/s12245-023-00509-w

**Published:** 2023-05-19

**Authors:** Henry Ashworth, Annie Lewis-O’Connor, Samara Grossman, Taylor Brown, Sadie Elisseou, Hanni Stoklosa

**Affiliations:** 1 Department of Emergency Medicine, Alameda Health System, Highland Hospital, Oakland, CA USA; 2 Department of Medicine, Brigham and Women’s Hospital, Boston, MA USA; 3 Department of Psychiatry, Boston Public Health Commission, Boston, MA USA; 4 Department of Emergency Medicine, Beth Israel Deaconess Medical Center, Boston, MA USA; 5 Department of Medicine, Harvard Medical School, Boston, MA USA; 6 HEAL Trafficking, Los Angeles, CA USA

**Keywords:** Trauma, Trauma-informed care, Safety, Quality improvement, Harm reduction, Quality of care, Psychological first aid

## Abstract

A patient’s current or previous experience of trauma may have an impact on their health and affect their ability to engage in health care. Every year, millions of patients who have experienced physically or emotionally traumatic experiences present to emergency departments (ED) for care. Often, the experience of being in the ED itself can exacerbate patient distress and invoke physiological dysregulation. The physiological reactions that lead to fight, flight, or freeze responses can make providing care to these patients complex and can even lead to harmful encounters for providers. There is a need to improve the care provided to the vast number of patients in the ED and create a safer environment for patients and healthcare workers. One solution to this complex challenge is understanding and integrating trauma-informed care (TIC) into emergency services. The federal Substance Abuse and Mental Health Service Administration’s (SAMHSA) six guiding principles of TIC offer a universal precaution framework that ensures quality care for all patients, providers, and staff in EDs. While there is growing evidence that TIC quantitatively and qualitatively improves ED care, there is a lack of practical, emergency medicine-specific guidance on how to best operationalize TIC. In this article, using a case example, we outline how emergency medicine providers can integrate TIC into their practice.

## Background

**Fig. 1:**
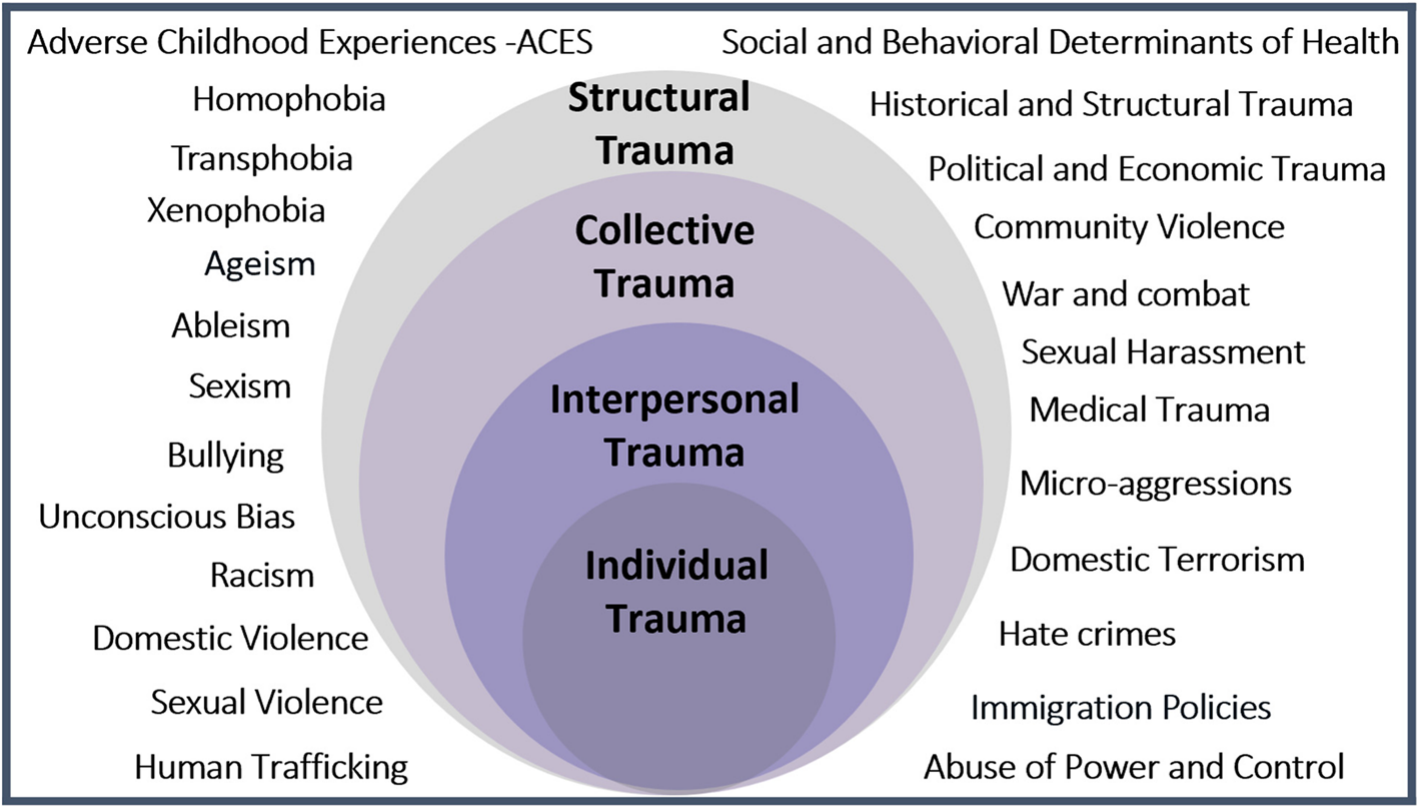
The socio-ecological model of trauma. This diagram of the ecological model of trauma shows the wide range of trauma that can be experienced across individual and collective levels (© Lewis-O’Connor, A. 2015 © Rittenberg, E 2015 © Grossman, S. 2015 UPDATED, April 2020, Feb 2022)

Emergency departments (EDs) across the USA treat millions of patients per year who suffer from trauma, defined as experiences that are physically or mentally harmful and have lasting adverse effects on mental, physical, social, or spiritual health [1–4]. While most think of “trauma” as physical injury, it can also include events or circumstances that stem from interpersonal, collective, and structural experiences such as racism and sexism. Trauma is universal and intersectional, affecting individuals across domains such as gender, race, and community, as demonstrated in the ecological model of trauma (Fig. 1) [5, 6]. Adverse childhood experiences (ACEs) are various traumatic exposures, including neglect, abuse, and/or household dysfunction, which occur before the age of 18. ACEs are extremely common, with over 60% of US adults reporting ACEs [4, 7–9]. We can therefore assume that the majority of ED patients have previously experienced trauma. Some studies suggest higher ED utilization among those with higher trauma burdens as measured by ACEs [10]. These numbers increase for those most impacted by collective traumas, as demonstrated in the higher numbers of ACEs for Black, Indigenous, Latinx, and LGBTQ + communities [7–9].

The impact from these traumatic experiences can be cumulative. ACEs have been shown to increase rates of heart disease, cancer, substance use disorder, and psychiatric illness in adulthood [1, 9]. In addition to affecting physical health, these experiences may result in changes to the nervous system. External stimuli that are either dangerous, perceived as dangerous, or reminiscent of past traumatic experiences can elicit a sympathetic reaction, often called dysregulation [10]. When patients experience this and become dysregulated, they can either act out and risk being labeled as “difficult” or “combative” or shut down and risk being labeled as “not engaging in care” [11]. Exposure to external stimuli that reminds patients of their past trauma, or repeated experiences of traumas themselves (e.g., ongoing exposure to structural racism), can result in long-term health impacts [7].

While it is common for patients to be combative on arrival to the ED, it is also true that 60% of people who have prior traumatic experiences seek care in the ED. Or many of those patients, the environment, and healthcare procedures can be triggering, and some may also find ED care itself to be traumatizing. Being in the ED’s chaotic, noisy environment and experiencing stigma, bias or other forms of oppression expressed in care delivery can cause patients to become dysregulated and react to real or perceived threats with maladaptive behaviors [11, 12]. Previous trauma can also erode a patient’s trust with the healthcare system, making it challenging for a patient to engage in receiving health care. Therefore, it is essential that efforts are made by healthcare providers to prevent re-traumatization in the ED [12].

Due to the prevalence of trauma and the impact of trauma on health, we have an important opportunity to improve care for millions of patients in the ED. One solution for improving care is trauma-informed care (TIC). TIC is a well-established, theoretical, and organizational framework for supporting trauma survivors that can be applied to create a safer environment for patients and healthcare professionals and improve both process and patient outcomes in the ED [12].

The framework for TIC was created in 1994 by the Substance Abuse and Mental Health Services Administration (SAMHSA), expanding upon prior trauma research to improve care provided to survivors of physical and sexual violence and substance use disorders. Its six core principles are crucial to improving care for all patients [2]. These principles are as follows: (1) safety; (2) trustworthiness and transparency; (3) peer support; (4) collaboration and mutuality; (5) empowerment, voice, and choice; and attending to (6) cultural, historical, and gender issues. Building the 6 principles of TIC into every encounter as a universal precaution allows patients to regain control that may have been lost in present or past traumatic experiences [11].

The field of TIC research in emergency medicine (EM) is growing, with both quantitative and qualitative data revealing that TIC improves patient care [12–17]. Quantitative studies have shown that implementing TIC education programs can reduce the number of patients subjected to physical restraints and the overall time patients spend in restraints in EDs [13] and inpatient settings [14]. Furthermore, studies have shown that TIC improves care for those who have experienced trauma ranging from ACEs [10] to sexual assault in adults [1, 18].

Recognizing the impact of TIC, in 2012, the US Attorney General National Task Force on Children Exposed to Violence called for all ED providers interacting with patients to be trained in TIC (20). Despite this call and growing evidence for the application of TIC to improve both subjective and objective patient outcomes, many EM physicians lack familiarity with and training on TIC [18–20]. TIC may play a role in improving quality healthcare delivery; thus, it is critical that all EM personnel, including clinicians, nurses, and nonclinical staff, learn practical applications of TIC.

Implementing TIC into practice should not be viewed as additional work; rather, a reframing of how health care is delivered by applying the core principles of TIC [21, 22]. To help inform EM practitioners, we outline specific suggestions for applying TIC clinically throughout a patient encounter. We also supply supplementary tables outlining specific TIC approaches providers can use in their encounters to empower patients, increase patient autonomy, and build trust. Such approaches can mitigate re-traumatization, promote patient autonomy, and ultimately craft a more meaningful encounter. Our hope is that this outline provides tools that ED providers can utilize when taking a patient history, conducting a physical examination, and performing routine procedures.

## Case presentation

Ms. Anaya Robinson is a 39-year-old woman who presents to the ED after a low-speed single motor vehicle crash. The triage notes state that she is complaining of head and arm pain. The initial assessment report notes that she appears “agitated” and is “guarded.”

EMS reports that the patient was driving less than 25 mph when the car veered off the road and struck a guardrail. On the scene, the patient had a Glasgow Coma Scale (GCS) of 15 with mild disorientation, stable vitals, and no complaints apart from moderate bruising to her right eye and a long laceration along her right forearm. There was minimal damage to the car, the air bag did not deploy, and a c-collar was placed on scene. In the ED, her vitals have continued to be stable.

### Taking a trauma-informed history of present illness

Given the high prevalence of trauma among all adults, TIC should be applied using a universal precaution approach in taking a patient’s history [21]. The history taking provides an opportunity to establish a relationship with the patient and set the tone for establishing a trauma-informed d encounter. Table 1 provides guidance as to how to build trust by engaging the six principles of TIC from the start of an encounter.

**Table 1:** Strategies for taking a trauma-informed patient history

Usual care approach	SAMHSA’s six principles	Trauma-informed care approach
• Seeks to ensure physical comfort • Assume that aggression or resistance is willful on the part of the patient. Does not consider hyper or hypo-arousal response (fight/flight/freeze) • May ask “why are you here?” “What brings you here today?”	**Safety**	• Assumes that any patient arriving to ED is responding from a hyper/hypo-arousal response (fight/flight/freeze), and as such is alert to cues for danger and may respond with aggression (fight), resistance (flight), or non-responsiveness (freeze) • Uses open-ended questions to explore ways to establish safety or threats to safety • “Is there anything I can do right now that would make you feel more comfortable?”
• Alerts patient of steps that will be taken, in the order they will happen. “I am here today to take a short history of what happened before you arrived in the ED. Do you mind telling me the events that led up to you coming here?” • Does not focus on building relationships or explaining roles or next steps. If called out of the room unexpectedly will note this and leave: “I am sorry I am being paged”	**Trust & transparency**	• Begins with provider name, role, time that will be spent with the patient and why, and next steps. “My name is ___, and my role is ___, I am here to learn more about you. It is likely that another provider will also come in and ask you these same questions, as each provider seeks to learn as much as they can about you. I will do my best to relay as much information as I can to the next person. Before we begin, do you have any questions for me about this visit?”
• Offers patient options for support after visit. “Would you like any resources about what happened to you? I have a sheet I can give you or I can refer you to a social worker who is knowledgeable in these things”	**Peer support**	• Offers the patient the option of discussing resources and waits for response before discussing. “Would you like me to connect you with anyone or any groups who have experienced similar events as you have?” • If seeking to engage a social worker, explain their role, and explain that the social worker is a reliable referral. Seek to create warm handoffs when possible
• Alerts patient that care received is on a team-based model. “We will fax a copy of your paperwork to your PCP, so they are updated”	**Collaboration & mutuality**	• Seeks to make concrete options available to patients about how they can be involved in their current care. “I would like to ensure that we all work as a team during your visit. If you have questions at any point, please ask them, including questions about what is going into your chart. We will also talk about your treatment plan together, so we both feel comfortable with next steps in your care”
• Offers patients a chance to ask questions and engages patients in the care encounter. “Now that I have explained what’s going to happen today, do you have any questions?”	**Empowerment, voice, & choice**	• Views the patient as an equal expert and collaborator in care and treatment planning and actively seeks to involve the patient. “You are the expert in your body – what do you think may have been missed thus far?”
• Does not usually ask about patient culture, gender orientation, or impact of historical issues. May make assumptions about race, ethnicity, gender, or background	**Cultural, gender, historical issues**	• Seeks to gain a holistic understanding of the patient, so that context of trauma is understood. Inquire out about their lived experiences, customs, and preferences. “Many patients have shared something about themselves, or their preferences for care- is there something we might specifically do for you to address your need” • Ensure a translator is available to collect an accurate history

The chaotic environment of the emergency department can make it challenging to establish the TIC principle of safety, given that patients often share rooms or are in hallways. Before beginning the interview, consider what resources can be utilized to help establish safety, including interview rooms, dividers, available staff, and patient visitors when appropriate. In Ms. Robinson’s case, you establish safety by knocking, announcing yourself, and asking permission to enter. When entering the room, you make sure to leave a clear exit for yourself and the patient. You establish trust and transparency by identifying yourself, stating your role in the patient’s care, and allowing other members of the team to do so as well. You ask the patient for their name and pronouns to support autonomy and gender inclusivity. If appropriate, we also recommend inviting the patients to share cultural identities in a manner that is inclusive such as “We invite all our patients to share something about themselves, or their preferences for care. Is there something we might specifically do for you to address your needs or get to know you in a more meaningful way.”

After doing so with Ms. Robinson, you learn that she prefers to go by Anaya, she identifies as an African American female, and her pronouns are she/hers. You provide anticipatory guidance for what will happen during this encounter, establish expectations on timing, and ask what major concerns she has. During the interview, Anaya notes that she was angry and was texting a friend when her car went off the road and hit the guardrail. Upon eliciting past medical history, you learn she has hypertension, and no other medical conditions. She has had no previous surgeries. She has no allergies, and her only medication is lisinopril. As you finish taking the brief history, you summarize what you heard from and ask her to correct or add any information. This iterative process gives Anaya the ability to clarify her story and empowers her to feel she is an equal and active participant in her care.

### Performing a trauma-informed physical examination

The physical examination has the potential to cause discomfort and distress, particularly when patients are feeling vulnerable, unsafe, or fearful. At its worst, the physical examination can be re-traumatizing by activating memories of prior trauma. The limited time, space, and privacy of the ED can impact a provider’s ability to conduct a trauma-informed physical examination. However, these barriers can be overcome using specific strategies and collaborating with the patient and the care team. Before starting the physical examination, build trust through transparency by explaining the rationale for the examination and asking the patient about their preferences (trust and transparency). During the examination, create psychological and physical safety by limiting touch to what is medically necessary (safety), empower the patient by asking for permission to proceed or to stop (empowerment, voice, and choice), and collaborate by adapting the examination to the needs of each patient (collaboration and mutuality) [20] using shared decision-making. Finally, after the examination, the provider should share relevant findings, inquire if the patient has questions, and communicate next steps. A list of practical strategies for a trauma-informed physical examination is shown in Table 2.

**Table 2:** Strategies for performing a trauma-informed physical examination

Usual care approach	SAMHSA’s six principles	Trauma-informed care approach
• Not taking the time to enhance physical and psychological safety in the room even with interruptions (i.e., not obtaining curtains for hallway beds) • Leaving the patient exposed when not clinically necessary. For example, exposing both sides of the chest when only one is being examined • Using phrases that may have been a part of a traumatic experience (i.e., “relax” or “calm down”) • Using language that may have a sexual connotation such as asking patients to complete an action “for me” such as “open your mouth for me”	**Safety**	• If a patient is in a waiting room or hallway bed, take extra time to move them to an exam room, or ensure curtains can be moved to their location • Throughout the exam, drape or cover the patient to maintain comfort and privacy. Use appropriate draping techniques to systematically expose and re-drape areas as they are being examined • Use language that is neutral, objective, and professional to direct the patients’ actions. “Push the right arm forward” or “Could you please lift the left breast”
• Beginning the exam without a patient understanding what is being examined or why. This is especially important during trauma evaluations as patients are often scared and disoriented • Leaving the exam room without updating the patient on pertinent findings from the physical examination • Using words such as “good” or “nice” to describe findings. “Everything looks good. I will be back in a few minutes to check in”	**Trust & transparency**	• Outline the overall steps of the exam and how long it should take • If the patient would like narration, narrate to the patient what you are examining and why. Recap the major findings at the end of the physical examination using neutral language such as “healthy” to describe findings
• Either letting a visitor stay in the examination room when inappropriate or removing a trusted support person without checking in with the patient	**Peer support**	• Determine with the patient if it would be appropriate to have their visitor in the room during the exam or if they would like a chaperone. Often, patients may not feel comfortable if this individual is already in the room, so it is best to ask when the patient is alone
• Performing actions for patients without consent such as untying a gown before beginning the examination • Asking politely for the patient to perform actions without eliciting the patient’s perspective. “Just breathe for me, okay?” • Asking patients if they are comfortable, but not giving an option to improve their level of comfort	**Collaboration & mutuality**	• Consider ways to incorporate patients into the exam. For example, have the patient guide the stethoscope under the breast when auscultating • Check in to see if anything can be done to make the patient more comfortable, i.e., “Would you feel more comfortable sitting up or resting back?”
• Asking if the patient is ready to start the exam without exploring if they have any questions or concerns • Not creating the space for a patient to speak up for when they are uncomfortable or if they have any concerns	**Empowerment, voice, & choice**	• Invite questions or concerns the patient may have before beginning the exam. “Do you have any questions or concerns I can address about this exam?” • Ask for permission before touching the patient, especially in sensitive areas such as the neck, chest, breast, genitals, or rectum
• Not adjusting to meet a patient’s cultural needs (e.g., having a female provider perform a pelvic exam) • Not using a translator or having someone who is not a certified translator explain the purpose of the exam or narrate the exam	**Cultural, gender, historical issues**	• If an all-male team, find a chaperone for a female patient who is presenting for assault or intimate partner violence. Can be addressed by asking, “Would you be comfortable with a male physician performing the exam today?” • Consider how culture may influence the perceived appropriateness of a provider’s touch • Utilize translator services when appropriate to ensure informed consent for the examination

Before beginning the examination, you state the rationale (why) and extent of the procedure to establish transparency and build trust with Anaya. You explain to Anaya that since she was in a motor vehicle crash, she will need a “full head to toe examination” to assure her well-being. After explaining this, you ask what concerns she has, invite her to ask questions, and inquire how you can maximize her comfort. This will help level the power dynamic in the room, empowering Anaya as a collaborator in her care. Anaya voices appreciation for your approach, stating she feels like the c-collar is choking her, and she wants to take it off immediately.

Using the Canadian C-spine rule, you discuss how you can test her range of motion to decide whether to remove the c-collar. As you step behind the head of the stretcher, you notice that Anaya tenses and starts to hyperventilate. It can be very challenging to notice patients’ sympathetic responses as they can be subtle and occur during actions that are very routine to providers. Important positive and negative physical signs to notice include tensing, flinching, freezing, sudden disengagement, sweating, darting eye movements, increased volume, or pressured speech. Changes in vitals such as heart rate, respiratory rate, and blood pressure can be useful if a patient is also on a monitor, but these do not always occur. Recognizing Anaya’s change in respiration as a sympathetic threat response, you pause and say, “I notice you’re breathing faster. Are you feeling okay?” She explains that she does not like being unable to see where you are and is now feeling more anxious. Moving to the side of the stretcher, you discuss with Anaya how you will stabilize her neck from the front while you clear her C-spine. You communicate the steps of your exam and explain how a nursing colleague will assist, so that Anaya remains engaged in the procedure. While holding the C-spine, the nurse removes the collar, and you clear her C-spine. You notice that Anaya’s breathing has slowed down, and she appears more relaxed.

After finishing the physical examination, you ask Anaya how you can adjust the stretcher to make her comfortable. Once she is settled, you review the main findings with Anaya and discuss the need to repair the laceration on her forearm. As you finish putting in other orders, you are called away to a rapid assessment. To remain transparent with Anaya, you share that you are being asked to assist with another patient and will return to repair the laceration when you can. You thank her for her patience.

### Performing a trauma-informed procedure

As procedures can be invasive and painful, they can be particularly challenging for individuals with a trauma history. Procedures that adhere to the guiding principles of TIC can mitigate re-traumatization. Trauma-informed approaches to procedures are similar to those recommended for physical examinations [22]. The priority before, during, and after the procedure is to help the patient feel physically and psychologically safe, build trust, provide choices, and seek to understand the patient’s concerns and preferences. While you are aware that a laceration repair is a standard and often simple procedure, it can nonetheless be distressing for patients. A list of advice and examples for performing a trauma-informed procedure can be seen below in Table 3.

**Table 3:** Strategies for performing trauma-informed care procedures

Usual care approach	SAMHSA’s six principles	Trauma-informed care approach
• Touching patients without asking • Holding down or restraining a patient’s extremity during the procedure • Starting the procedure before the patient is ready to proceed and/or properly anesthetized	**Safety**	• Acknowledge that while the procedure is routine to you, it is not routine for the patient • Achieve pain relief before starting procedures • Instruct the patient how to position their extremity without restraining the extremity • Stop the procedure if instructed by the patient
• Not announcing procedure steps before proceeding • Assurances that do not validate a patient’s feelings (i.e., it is just a simple procedure) • Questioning the patient’s level of pain, history, choices • Providing prescriptive care and treatment, rather than offering choices	**Trust & transparency**	• Partner with patients during the procedure and identify ways the patient best copes • Respecting the patient’s preferences without judgment • Explain each step of the procedure including equipment used • Provide anticipatory guidance for sounds and sensations (e.g., burn from lidocaine) if desired
• The patient is unable to communicate patient preference to the care team • The team fails to offer or denial of the presence of a support person	**Peer support**	• Inquiring about supports available to the patient • Closed-loop communication among team members around patient’s preferences • Offer the patient to have a chaperone or support person present (consider virtual options as well)
• Failure to offer or honor adjustments requested by patients • Failure to communicate the results of the procedure • Lack of coordination among services if consulting services are involved	**Collaboration & mutuality**	• Promote shared decision-making • Inquire about how previous healthcare procedures have been tolerated • Make adjustments based on patient preference (i.e., patient positioning, support person, music) • Discuss results of procedure with patient at conclusion of procedure
• Failure to inform patients of the option to stop or take a break at any time • Failure to offer adjustment and choices	**Empowerment, voice, & choice**	• Increase proactive, shared decision-making during the procedure • Involve the patient in the procedure if they choose (i.e., hold drape, self-insert speculum, position so they can watch) • Acknowledging the patient’s strengths and attributes • Empower patients to request to slow down, pause, or stop at any time • Allow choices where possible (i.e., position of comfort, music, support person, narration)
• Having preconceived assumptions about certain populations, for example, bias against people with substance use disorders, mental health challenges, communities of color, and LGBTQIA • Not reflecting on and taking measures to address one’s unconscious bias	**Cultural, gender, historical issues**	• Seek to increase self-awareness of unconscious bias and stigma, including bias related to pain thresholds and pain management • Understand that patient reactions may be related to past trauma including medical trauma and experiences with structural systems

In preparing to suture, you explain the procedure to Anaya, including the instruments and medications that will be used. You explain that the local anesthetic typically burns when initially injected, but that this sensation is temporary. You specifically ask what questions she has, clarify information, and discuss how to best engage Anaya in the process of the procedure. By moving through this process, Anaya feels more of a sense of trust and is empowered to speak up in case she begins to feel uncomfortable again as she did during the physical examination.

Collaborating with Anaya, you may ask: How might we best support her during this procedure? What position would be most comfortable, for example, sitting up so she can see the procedure or reclining with a limited view of the procedure? How has she done with procedures in the past? Anaya shares that she prefers to sit up so that she can see what is happening, and that she fears pain. She recalls a prior laceration repair when she did not have enough anesthetic. You thank her for sharing a difficult experience and ensure that you will not begin suturing until you assess with her that the area is numb.

You tell Anaya that she can request you to stop or slow down at any point during the procedure. As some patients prefer step-by-step details during a procedure and others may prefer you swiftly proceed, you ask her how much information she would like to hear. She states that she prefers to know how much time is left before the procedure will be completed, but not each step. She also asks if she can listen to music on her phone to which you answer affirmatively.

After several sutures are complete, Anaya states that she feels nauseous, and that she may pass out. You notice she is clenching her fist, and that her heart rate has increased on the monitor. Noticing this sympathetic response, you pause and encourage her to take a deep breath. She states that she cannot feel any pain but is starting to become overwhelmed and would like a minute. You state you will give her some time and return in several minutes, to which she agrees. You return and reassess how she is feeling. Anaya states her forearm is still numb and confirms that she is ready to proceed with the remaining sutures. Anaya thanks her care team for being so patient and compassionate with her.

### Completing a trauma-informed discharge

Over the last few hours, the ED has hit a new critical level of patients, and the average wait time has increased by 2 h. You are considering discharging Anaya, but in reflecting on the prevalence of trauma and Anaya’s reactions during the patient encounter, you feel that Anaya may have other underlying needs. Since she does not share other needs, you instead provide a general list of local community resources for social work and mental health in her discharge instructions, after inquiring if she can safely take this information with her. After reviewing proper care for her stitches, you briefly review these resources, stating, “I'd like to share these resources that are available to all of our patients, in case they may be helpful to you or a loved one. Is this information you would like to take with you?” Anaya responds in the affirmative; you assure this information is included in her discharge instructions.

## Discussion

The ED is a healthcare setting with a uniquely high prevalence of patients who have experienced complex physical, mental, and social traumas [23]. While TIC was initially developed as an organizational framework in which to care for trauma survivors, it is now conceptualized as a universal approach that should be applied to the care of all patients [2]. TIC has become routine care in fields such as pediatrics, and research has shown that TIC can improve patient experiences and objective outcomes [14, 15, 24]; however, subspecialties like EM lack adequate TIC training [15, 19, 25]. The case and summary tables presented in this paper provide concrete examples of how ED providers can improve patient care using trauma-informed principles in starting a patient encounter, conducting a physical examination, performing a procedure, and completing a discharge.

The examples provided are not exhaustive and offer only a brief overview of providing TIC. Practicing TIC is not formulaic — it is a fundamental reframing of how care is delivered. It is a style of practicing clinical medicine that focuses on creating a safe and empowering environment for patients in which providers and staff can also thrive. This article focuses specifically on how TIC can inform patient-provider interactions in the ED. In addition, TIC can be used to inform interprofessional communication, medical education [26], department leadership [27], and organizational structure [28]. While TIC has broad applications, we would also like to acknowledge that TIC is not the only solution for addressing individual and structural issues in the ED, yet it synergizes well with health equity and racial justice initiatives.

Caring for patients who have experienced trauma can elicit personal feelings from the clinical team. Dr. Kimberg provides a framework for clinicians that acknowledges personal reactions and promotes self-care (29). The four Cs highlight the following: (1) staying calm and paying attention to how you are feeling, noting that you may need to calm yourself in ways that promote calmness for yourself, your patients, and your colleagues; (2) contain history details inquiry about information that impacts the plan of care; (3) care for self and patient using self-compassion, cultural humility, and self- awareness about unconscious bias and stigma; and lastly, (4) cope — focusing on ways you cope and how patients best cope, promoting interventions that build resilience in self and supporting patient’s efforts in this regard.

Future work is needed to develop TIC curricula for undergraduate medical education, graduate medical education, and continuing medical education [29, 30]. This includes the need to develop national TIC educational competencies and clinical practice standards [26]. TIC training can serve as a foundation for providing high-quality, patient-centered care, from the individual to the structural levels.

As EDs care for an increasing number of patients with complex and intersectional trauma, frameworks such as TIC are needed to create safer environments for EM patients and healthcare professionals. TIC is reframing how care is provided to create more thoughtful, therapeutic relationships with patients. As such, by incorporating TIC as a critical tool, we may achieve better outcomes, for ourselves and the communities who come to us for care.

## Data Availability

Data sharing is not applicable to this article as no datasets were generated or analyzed during the current study.

## Abbreviations

ACEs: Adverse childhood experiences
ED: Emergency department
EM: Emergency medicine
GCS: Glasgow Coma Scale
LGBTQIA: Lesbian, gay, bisexual, trans, queer, intersexual, and asexual
SAMHSA: Substance Abuse and Mental Health Service Administration
TIC: Trauma-informed care
